# Clinical significance of transjugular liver biopsy in acute liver failure – a real-world analysis

**DOI:** 10.1186/s12876-024-03350-4

**Published:** 2024-08-08

**Authors:** Bahar Nalbant, Thorben Pape, Andrea Schneider, Benjamin Seeliger, Paul Schirmer, Benjamin Heidrich, Richard Taubert, Heiner Wedemeyer, Henrike Lenzen, Klaus Stahl

**Affiliations:** 1 Department of Pneumology and Infectious Diseases, Hannover Medical School, Hannover, Germany; 2 Department of Gastroenterology, Hepatology, Infectious Diseases and Endocrinology, Hannover Medical School, Carl-Neuberg-Str.1, 30625 Hannover, Germany; 3 Member of the European Reference Network On Hepatological Diseases (ERN RARE-LIVER), Hannover, Germany

**Keywords:** Acute liver failure, Transjugular liver biopsy, Liver biopsy, Liver transplant free survival

## Abstract

**Background:**

Histopathological characterization obtained by transjugular liver biopsy (TJLB) may theoretically contribute to clarification of the exact aetiology of acute liver failure (ALF). It's unclear whether the histopathological information from TJLB, due to the small specimen size, significantly contributes to diagnosing ALF causes, guiding therapy decisions, or predicting overall prognosis. This retrospective study aimed to analyse safety and clinical significance of TJLB in patients with ALF.

**Methods:**

This retrospective, monocentric study investigated safety and efficacy of TJLB in patients with ALF over a ten-year period at a tertiary care transplant-center. The predictive value of various clinical and laboratory characteristics as well as histopathological findings obtained by TJLB on 28-day liver-transplant-free survival were evaluated by calculating uni- and multivariate Cox-proportional hazard regression models. Additional univariate logistic regression analyses were performed to explore the influence of degree of intrahepatic necrosis on the secondary endpoints intensive-care-unit (ICU) admission, need for endotracheal intubation, renal replacement therapy and high-urgency listing for LTX.

**Results:**

A total of 43 patients with ALF receiving TJLB were included into the study. In most cases (*n* = 39/43 cases) TJLB confirmed the initially already clinically presumed ALF aetiology and the therapeutic approach was unchanged by additional histological examination in the majority of patients (36/43 cases). However, in patients with a high suspicion for aetiologies potentially treatable by medical immunosuppression (e.g. AIH, GvHD), TJLB significantly influenced further treatment planning and/or adjustment. While the degree of intrahepatic necrosis showed significance in the univariate analysis (*p* = 0.04), it did not demonstrate a significant predictive effect on liver transplant-free survival in the multivariate analysis (*p* = 0.1). Only consecutive ICU admission was more likely with higher extent of intrahepatic necrosis (Odds ratio (OR) 1.04 (95% CI 1–1.08), *p* = 0.046).

**Conclusions:**

Performance of TJLB in ALF led to a change in suspected diagnosis and to a significant change in therapeutic measures only in those patients with a presumed high risk for aetiologies potentially responsive to immunosuppressive therapy. Clinical assessment alone was accurate enough, with additional histopathological examination adding no significant value, to predict overall prognosis of patients with ALF.

## Background

Acute liver failure (ALF) occurs unexpectedly, has a rapid onset and affects previously healthy individuals [1]. The prognosis of patients with ALF varies substantially and depends on various factors such as the patient age, the degree of encephalopathy, the extent of extraintestinal multi-organ failure, as well as the underlying aetiology [2]. ALF is characterized by common clinical characteristics, namely elevated liver enzymes combined with encephalopathy and coagulopathy [3]. Therefore, the diagnosis of ALF is primarily based on clinical symptoms and routine blood tests [4]. Liver histology, obtained by biopsy, remains one of the most important diagnostic methods in the workup of unclear hepatopathy [5] and can be performed in various ways, including percutaneous, laparoscopic and transjugular approaches [6]. Histological features obtained by liver biopsy may aid clinicians in deciding on liver transplantation for these patients [7]. The transjugular liver biopsy (TJLB) appears to be safer compared to percutaneous liver biopsy, particularly in cases of haemostatic impairment or advanced liver cirrhosis [8]. Consequently, the most common indications for TJLB are ascites and thrombocytopenia [9]. However, despite these potential benefits, there is uncertainty regarding benefit and safety of TJLB in patients with ALF [10]. Especially, it is unclear if the histopathological information obtained by performing TJLB, given the rather small specimen size obtained in a potentially high degree of intrahepatic necrosis, is able to significantly contribute to diagnosis of ALF aetiology, therapeutic decision making and prediction of overall prognosis. At the same time, performance of TJLB is resource intensive as the patient has to be transported from a high-dependency unit to a radiology suite for fluoroscopic guidance. This retrospective real-life study aimed to analyse safety and clinical significance of TJLB in patients with ALF.

## Materials and methods

### Selection and participation in the study

**Fig. 1:**
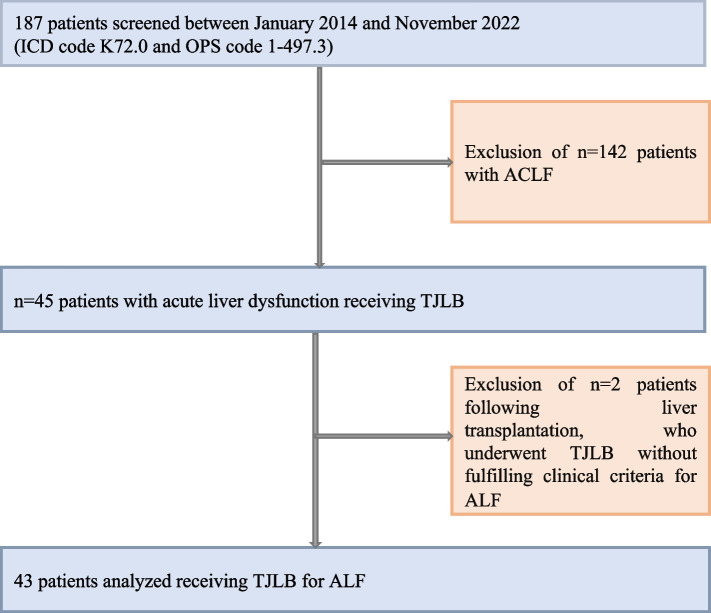
Study inclusion. One hundred eighty-seven patients with acute liver dysfunction between January 2014 and November 2022 were screened. 43 patients with acute liver failure undergoing TJLB were included for final analysis. 43 TJLB interventions were successful and included into the study. In total, 147 patients with acute-on-chronic liver failure were excluded, along with two additional patients who underwent elective biopsy as per the liver transplantation protocol despite not having acute liver failure

This retrospective, monocentric study investigated safety and efficacy of TJLB in patients with ALF over a ten-year period at a tertiary care university hospital (Fig. 1). A total of 187 patients with acute liver dysfunction (German ICD code K72.0) receiving TJLB (German OPS code 1–497.3) were initially screened. 142 cases of acute-on-chronic liver failure were excluded. Two of the patients underwent a biopsy following liver transplantation without fulfilling clinical criteria for ALF. Two patients with acute alcoholic steatohepatitis (without cirrhosis) have been included into this analysis despite acute steatohepatitis being per definition not ALF, since clinical presentation and phenotype of these two individual cases did not differ from other patients with ALF. Eventually, 43 patients with ALF undergoing TJLB were included for final analysis. Time point of study inclusion was the day of obtaining TJLB (= baseline). All patients were followed up until day 28, hospital discharge or death. The ethical committee of Hannover Medical School (Nr. 11325_BO_K_2024) approved the protocol and written informed consent was obtained from participants or authorized representatives. The study was performed in accordance with the ethical standards laid down in the 1964 Declaration of Helsinki and its later amendments.

### Transjugular liver biopsy

TJLB was initiated in patients with ALF as an additional diagnostic method to supplement the clinical and laboratory presentation when transcutaneous biopsy was not feasible due to manifest coagulopathy. This minimally invasive procedure allows tissue to be removed from the liver under local anaesthesia and was performed as described previously [11]. In short, a catheter sheath was first inserted into the internal jugular vein and then a guidewire was introduced in a hepatic vein under fluoroscopic guidance. A transjugular biopsy needle was then placed over the guidewire into the hepatic vein and a tissue sample was taken (Cook-Medical, Limerick, Ireland). The hepatic venous pressure gradient (HVPG), a measurement of the pressure difference between the portal venous system and the inferior vena cava [12], was determined in a subset of patients (*n* = 19). The biopsy specimen was then sent to the in-house pathology department for rapid examination of the tissue. The Ishak score, summarizing various histologic features in the liver specimen, including inflammation, fibrosis and necrosis [13] was determined in all and the degree of intrahepatic necrosis (ranging from 0 to 100%) in the majority (*n* = 31) of patients, respectively.

### Endpoints

This study aimed at exploring the potential impact of TJLB on:

- diagnosis of ALF aetiology
- therapeutic decision making and
- prediction of prognosis

in patients with ALF. Therefore, potential change of presumed ALF aetiology and consecutive therapeutic approaches by the results of TJLB were retrospectively evaluated from chart records as clinical reasoning and decision making were compared before- and following TJLB. The primary endpoint was liver-transplant-free survival until day 28 from study inclusion. The predictive value of various clinical and laboratory characteristics as well as histopathological findings, e.g. Ishak Score and degree of intrahepatic necrosis, on the primary were evaluated. Further, influence of degree of intrahepatic necrosis on further secondary endpoints, e.g. intensive care unit (ICU) admission, need for endotracheal intubation, renal replacement therapy and high urgency (HU) listing for liver transplantation (LTX) was determined.

### Data collection

All personal patient data were pseudonymized before further analysis. Various demographic and clinical characteristics of patients with ALF undergoing TJLB were evaluated at the time point of obtaining TJLB (= baseline). Data were collected using electronic patient records, including the SAP and m.life patient data monitoring system (PDMS). The Model for the Endstage of Liver Disease (MELD) [14] and the BiLE Scores [15] were calculated at time of TJLB. The BiLE score consists of the three parameters blood bilirubine- and lactate concentration as well as the presumed most likely aetiology of acute liver failure. In a large German patient cohort of acute liver failure, ROC analysis revealed a better performance of the BiLE score when compared with bilirubin, lactate, MELD and SAPS-III score and a cut-off value above 6.9 was highly predictive for consecutive liver transplantation or death [15].

### Statistical analysis

Data were analysed using GraphPad Prism (Version 9.0, GraphPad Software, La Jolla, CA) and IBM SPSS Statistics (Version 27.0, IBM Corp., Armonk, NY). GraphPad Prism software (version 9.0, GraphPad Software, La Jolla, CA) was used to create graphs. Categorical variables are represented by number (n) and percentage (%), while for continuous variables they are expressed as median. The normal distribution was checked using the D'Agostino-Pearson omnibus normality test and the Shapiro–Wilk normality test. The predictive value of various clinical and laboratory characteristics as well as histopathological findings obtained by TJLB on the primary endpoint, 28-day liver-transplant-free survival, were evaluated by calculating uni- and multivariate Cox-proportional hazard regression models. All variables with a p-value of below 0.1 in the univariate analysis were eligible for consecutive inclusion into the multivariate analysis. Additional univariate logistic regression analyses were performed to explore the influence of degree of intrahepatic necrosis on the secondary endpoints ICU admission, need for endotracheal intubation, renal replacement therapy and HU listing for LTX. Survival was visualized using Kaplan–Meier plots and analysed using the log-rank test. All reported *p*-values are two-sided unless indicated otherwise; *p*-values < 0.05 were considered statistically significant.

## Results

### Cohort characterization at time of biopsy

A total of 43 patients with ALF receiving TJLB were included into the study (Table 1). The median (interquartile range (IQR)) age was 55 (37–64) years and the majority of 30 patients (69.8%) were female. At time of TJLB, all patients had manifest hepatic encephalopathy (HE), with grade I HE in 16 (37.2%), grade II HE in five (11.6%) and grade III HE in 22 (51.2%) patients, respectively. At this time, 17 (39.5%) patients were already admitted to the ICU, one patient (2.3%) was invasively ventilated, five patients (11.6%) were receiving renal replacement therapy (RRT) and ten patients (23.2%) therapeutic plasma exchange (TPE). The median (IQR) MELD-Score was 30 (27–35) points. Autoimmune hepatitis (AIH) antibodies were detectable before biopsy obtainment in the majority of patients, e.g. antinuclear antibodies (ANA) in 26 (60.4%) and smooth-muscle (SMA) in 28 (65.1%) patients, respectively. Median ANA and SMA-titers were 1:160 and 1:80, respectively.

**Table 1:** Demographic and clinical parameters at time of transjugular liver biopsy

Category	N (%)/Median (IQR)
Age, years	55 (37–64)
BMI, kg/m^2^	24.4 (21.9–28.6)
Sex	
female	30 (69.8)
male	13 (30.2)
HE	
Grade I	16 (37.2)
Grade II	5 (11.6)
Grade III	22 (51.2)
Grade IV	0
ICU Admission	17 (39.5)
Invasive Ventilation	1 (2.3)
RRT	5 (11.6)
Vasopressor therapy	3 (7)
Therapeutic Plasma Exchange	10 (23.2)
INR	2.2 (1.9–3.4)
AST, U/l	1227 (452–1682)
ALT, U/l	1301 (408–2601)
Bilirubin, µmol/l	342 (186–446)
Creatinine, µmol/l	76 (59–161)
MELD, points	30 (27–35)
Lactate, mmol/l	2.5 (1.7–3.4)
Ammonia, µmol/l	60 (46–73)
Factor V, %	38 (19.5–48.2)
Autoimmune-Hepatitis Antibodies	
ANA positive	26 (60.4)
ANA Titer	1:160 (1:80–1:160)
SMA	28 (65.1)
SMA Titer	1:80 (1:80–1:160)
LKM	6 (14)
LKM Titer	1:160
Viral hepatitis diagnostic	
HAV-IgM	1 (2.3)
HBsAg	2 (4.6)
HEV-DNA	1 (2.3)

Displayed are demographics, clinical characteristics and laboratory parameters of the whole patient cohort at the time of liver biopsy. Values are presented as median (25% to 75% interquartile range) or if categorical as numbers and percentages

*Abbreviations*: *ANA* Antinuclear antibodies, *BMI* Body mass index, *HAV-IgM* Hepatitis A IgM antibody, *HBs-Ag* Hepatitis B surface antigen, *HEV-DNA* Hepatitis E DNA (PCR), *HU* High urgency, *ICU* Intensive Care Unit, *LKM* Liver-kidney microsomal antibodies, *LTX* Liver transplantation, *RRT* Renal replacement therapy, *SMA* Smooth-muscle antibodies

Within 28 days following TJLB, 14 (32.6%) patients died. 15 (34.9%) patients, fulfilling Kings-College criteria, were HU listed for LTX and 14 (32.6%) received a graft. The one patient listed for HU-LTX but not getting transplanted was admitted with acute liver failure fulfilling Kings-College criteria. During the course of treatment, she developed a severe systemic inflammatory response syndrome, which resulted in cardiac arrest and unsuccessful cardiopulmonary resuscitation two days after transplant listing.

### Procedural and safety characteristics of transjugular liver biopsy

TJLB procedure related complications occurred in none of the patients. The technical success rate was 100%. The median (IQR) size of the TJLB specimen was 1.3 (0.3–3) cm. The biopsy was usable for further histopathological examination in all but two patients. In one patient biopsy was not representative, in the other too much intra-hepatic necrosis was present for further histopathological examination. The median (IQR) radiation dose applied was 1477 (551–3828) cGy/cm^2^.

### Impact of histopathological results obtained by transjugular liver biopsy for diagnosis of aetiology and further therapeutic approach

The most likely assumed aetiology of ALF and the therapeutic approach before- and after obtaining histopathological results by TJLB were compared in all patients by re-examining chart records (Table 2). 23 (53.4%) patients had suspected drug-induced liver failure with no change in diagnosis or therapeutic approach following biopsy. Five (11.6%) cases were initially categorized as cryptogenic, of which a definitive diagnosis was found in three of these patients and therapeutic measures were changed in only one (patient with histopathological diagnosis of AIH). In the first two patients the biopsy results were compatible with a potential diagnosis of drug-induced acute liver failure, an aetiology not initially suspected by past medical history at admission. In both of these two patients repeated past history taking after performing TJLB revealed that they had undergone been taking increased amounts of analgesics due to breast reconstruction a few weeks prior and intercostal neuralgia, respectively, facts that have not been admitted on admission. In the third patient, who was initially treated for cryptogenic acute liver failure, the biopsy showed a significant accumulation of plasma cells. Laboratory tests, that were finalized only a few days after receiving the biopsy results, detected specific autoantibodies, compatible with autoimmune hepatitis. Viral hepatitis was confirmed as the presumed aetiology in four (9.3%) patients with no change in the consecutive therapeutic measures. High suspicion for AIH as underlying aetiology was present in five (11.6%) patients and this diagnosis was confirmed by TJLB in four of these. Immunosuppression was started before TJLB in three of these patients and was continued as long-term immunosuppression in both following then definitive diagnosis of AIH. In one presumed AIH aetiology TJLB could not confirm AIH but suggested a more likely drug induced cause and no immunosuppressive treatment was started. In another presumed AIH aetiology, histology was generally compatible with AIH, but hepatitis resolved without immunosuppressive therapy, which might have been in line with the new term of drug-induced autoimmune like hepatitis [16]. In two patients with sickle cell crisis associated ALF vaso-occlusive aetiology was presumed and this could be confirmed in both by histology, indicating repetitive exchange transfusions. Hepatic manifestation of graft versus host disease (GvHD) needed histologic confirmation in one patient, then indicating high dose immunosuppression.

**Table 2:** Significance of histopathological biopsy for clinical diagnosis and therapeutic approach

Most likely diagnosis before biopsy	Histopathological diagnosis	Diagnosis changed by biopsy	Therapeutic approach influenced by biopsy	Therapeutic approach before biopsy	Therapeutic approach after therapy	Outcome
autoimmune	drug-induced	**Yes**	**Yes**	supportive therapy	supportive therapy	deceased
autoimmune	autoimmune	No	**Yes**	immunosuppression	continuation of long-term immunosuppression	LTX
autoimmune	autoimmune	No	**Yes**	immunosuppression	continuation of long-term immunosuppression	LTX
autoimmune	autoimmune	No	**Yes**	immunosuppression	continuation of long-term immunosuppression	LTX
autoimmune	autoimmune	No	No	supportive therapy	no immunosuppression due to spontaneous recovery	decreased
cryptogenic	autoimmune	**Yes**	**Yes**	supportive therapy	immunosuppression	LTX
cryptogenic	drug-induced	**Yes**	No	supportive therapy	supportive therapy	deceased
cryptogenic	drug-induced	**Yes**	No	supportive therapy	supportive therapy	deceased
graft-versus-host disease	graft-versus-host disease	No	Yes	immunosuppression	continuation of long-term immunosuppression	deceased
vaso-occlusive (sickle cell crisis)	vaso-occlusive (sickle cell crisis)	No	No	exchange transfusion	continuation of exchange transfusion	alive w/o LTX
vaso-occlusive (sickle cell crisis)	vaso-occlusive (sickle cell crisis)	No	No	exchange transfusion	continuation of exchange transfusion	LTX
severe acute alcoholic steatohepatitis	steatohepatitis	No	No	supportive therapy	supportive therapy	alive w/o LTX
severe acute alcoholic steatohepatitis	steatohepatitis	No	No	supportive therapy	supportive therapy	alive w/o LTX
cryptogenic	unclear	No	No	supportive therapy	supportive therapy	deceased
cryptogenic	unclear	No	No	supportive therapy	supportive therapy	alive w/o LTX
drug-induced	drug-induced	No	No	supportive therapy	supportive therapy	LTX
drug-induced	drug-induced	No	No	supportive therapy	supportive therapy	LTX
drug-induced	drug-induced	No	No	supportive therapy	supportive therapy	alive w/o LTX
drug-induced	unclear	No	No	supportive therapy	supportive therapy	LTX
drug-induced	drug-induced	No	No	supportive therapy	supportive therapy	alive w/o LTX
drug-induced	drug-induced	No	No	supportive therapy	supportive therapy	alive w/o LTX
drug-induced	drug-induced	No	No	supportive therapy	supportive therapy	decreased
drug-induced	drug-induced	No	No	supportive therapy	supportive therapy	decreased
drug-induced	drug-induced	No	No	supportive therapy	supportive therapy	alive w/o LTX
drug-induced	drug-induced	No	No	supportive therapy	supportive therapy	alive w/o LTX
drug-induced	drug-induced	No	No	supportive therapy	supportive therapy	LTX
drug-induced	drug-induced	No	No	supportive therapy	supportive therapy	deceased
drug-induced	drug-induced	No	No	supportive therapy	supportive therapy	LTX
drug-induced	drug-induced	No	No	supportive therapy	supportive therapy	alive w/o LTX
drug-induced	drug-induced	No	No	supportive therapy	supportive therapy	alive w/o LTX
drug-induced	drug-induced	No	No	supportive therapy	supportive therapy	alive w/o LTX
drug-induced	drug-induced	No	No	supportive therapy	supportive therapy	decreased
drug-induced	drug-induced	No	No	supportive therapy	supportive therapy	decreased
drug-induced	drug-induced	No	No	supportive therapy	supportive therapy	alive w/o LTX
drug-induced	drug-induced	No	No	supportive therapy	supportive therapy	LTX and deceased
drug-induced	drug-induced	No	No	supportive therapy	supportive therapy	LTX
drug-induced	drug-induced	No	No	supportive therapy	supportive therapy	alive w/o LTX
drug-induced	drug-induced	No	No	supportive therapy	supportive therapy	LTX and deceased
HAV infection	virus infection	No	No	supportive therapy	supportive therapy	alive w/o LTX
HBV infection	too much intrahepatic necrosis	No	No	antiviral therapy	antiviral therapy	LTX and deceased
HBV infection	virus infection	No	No	antiviral therapy	antiviral therapy	alive w/o LTX
HEV infection	virus infection	No	No	antiviral therapy	antiviral therapy	alive w/o LTX
pregnancy-induced	pregnancy-induced	No	No	supportive therapy	supportive therapy	alive w/o LTX

Displayed are the as most likely assumed aetiology of ALF and the therapeutic approach before- and after obtaining histopathological results by TJLB were compared in all patients by re-examining chart records of the whole patient cohort at the time of liver biopsy. The change in diagnosis or alteration of the therapeutic approach was indicated as yes/no responses

*Abbreviations*: *HAV* Hepatitis A virus, *HBV* Hepatitis B Virus, *HEV* Hepatitis E Virus

In summary, in most cases (*n* = 39/43 cases) TJLB confirmed the initially already clinically presumed ALF aetiology and the therapeutic approach was unchanged by additional histological examination in the majority of patients (37/43 cases). However, in patients with a high suspicion for aetiologies potentially treatable by medical immunosuppression (e.g. AIH, GvHD), TJLB significantly influenced further treatment planning and/or adjustment in all of these patients.

### Predictors of liver-transplant free survival

**Fig. 2:**
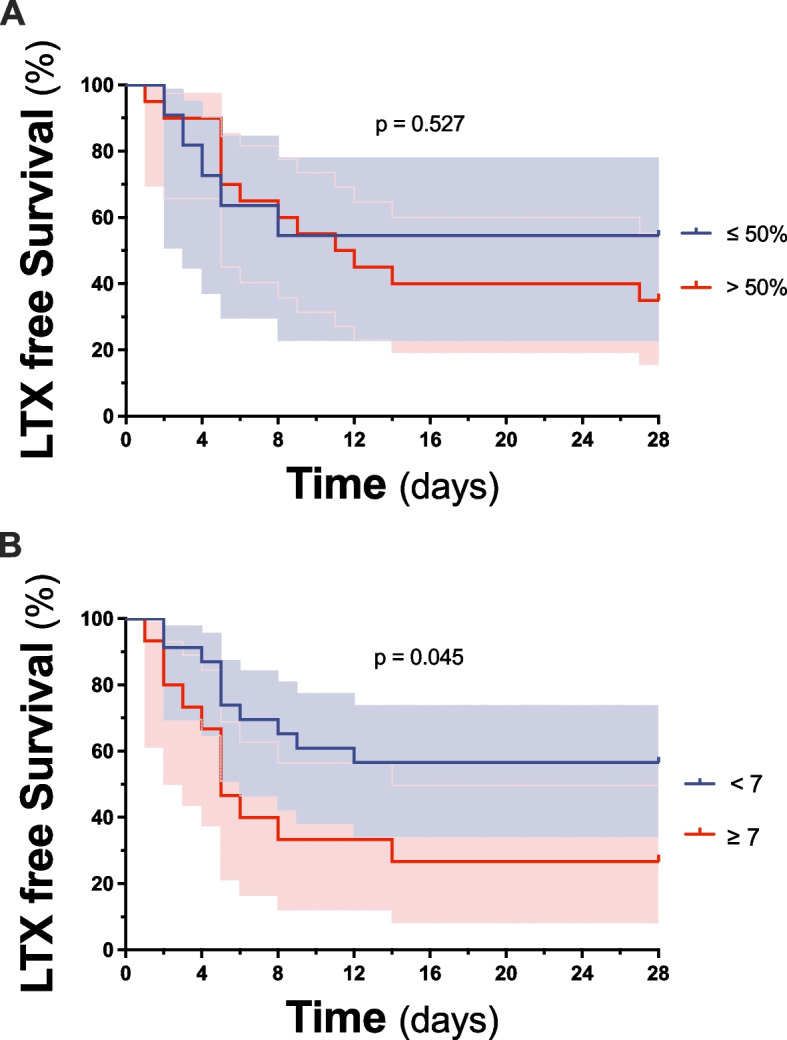
Prediction of liver transplant free survival by histopathological degree of necrosis. Influence of degree of intrahepatic necrosis on the LTX free survival () and effects on the grade of Bile Score of the LTX free survival (**B**). The results are shown as Kaplan–Meier graphs

The predictive value of various clinical and laboratory characteristics at the time of TJLB as well as histopathological findings obtained by TJLB, e.g. the Ishak Score categories and the degree of intrahepatic necrosis, on liver transplant-free survival were evaluated (Table 3). In the univariate analysis, the MELD Score (*p* = 0.06), blood lactate concentration (*p* = 0.02), pH (*p* = 0.06), the presence of HE greater than I° (*p* = 0.08), the BiLE Score (*p* = 0.002) and the need for vasopressor therapy (*p* =  < 0,01) were associated with liver-transplant free survival. None of the Ishak subscore categories showed a predictive value on liver-transplant free survival. While the majority of 31 patients (72%) had significant intrahepatic necrosis according to the Ishak score, the exact histopathological degree of intrahepatic necrosis in the TJLB specimen varied substantially in individual patients (Median (IQR): 60 (10–90)). While the degree of intrahepatic necrosis showed significance in the univariate analysis (*p* = 0.04), it did not demonstrate a significant predictive effect on liver transplant-free survival in the multivariate analysis (*p* = 0.08). In fact, the BiLE Score was the only parameter at the time of TJLB performance, that had a significant effect on transplant free survival (Hazard Ratio (HR) 1.2 (95% Confidence Interval (CI) 1.06–1.3), *p* = 0.002). Patients with acute intrahepatic necrosis of below and above 50% of the liver parenchyma had comparable liver-transplant free survival rates (Hazard Ratio (HR) 1.38 (95% Confidence Interval (CI) 0.5–3.9), *p* = 0.527, Fig. 2A). In contrast, a previously suggested cut-off value for the BilE Score [15] determined at the time of TJLB procedure, sufficiently predicted later liver-transplant free survival (Hazard Ratio (HR) 2.65 (95% Confidence Interval (CI) 1.02–6.88), *p* = 0.045, Fig. 2B).

**Table 3:** Predictors of liver transplant-free survival at time of transjugular liver biopsy

		**univariate**			**multivariate**	
	**HR**	**95% CI**	**p**	**HR**	**95% CI**	**p**
MELD Score, points	1,08	1 – 1,17	0,06	1	0,89 – 1,12	0,92
Lactate, mmol/l	1,15	1,02 – 1,3	0,02	0,56	0,29 – 1,06	0,08
pH	0,01	0,00 – 1,4	0,07	0	0,00 – 16,78	0,2
Ammonia, µmol/l	0,99	0,96 -1	0,5			
Factor V, %	1	0,97 – 1,03	1			
LDH, U/l	1	0,99—1	0,17			
HE greater than I°	2,31	0,92 – 5,9	0,08	1,08	0,30 – 3,81	0,9
BiLE Score, points	1,2	1,06 – 1,3	**0,002**	1,52	0,99 – 2,33	0,053
ICU admission	1,7	0,8 – 3,8	0,2			
Invasive Ventilation	0,05	0 – 1110	0,6			
RRT	0,65	0,16 – 2,75	0,5			
Vasopressor therapy	6	1,6 – 20	< 0,01	14	0,27 – 713,41	0,19
HVPG, mmHg	1	0,9 – 1,3	0,3			
Ishak-Score						
Ishak A	1,14	0,78 – 1,7	0,5			
Ishak B	1,3	0,9 – 1,8	0,17			
Ishak C	1	0,6– 1,7	0,9			
Ishak D	1,5	0,9 – 2,5	0,14			
Ishak F	0,8	0,5 – 1,1	0,2			
Intrahepatic necrosis, %	1,03	1 – 1,06	**0,04**	1,03	1– 1,07	0,1

Displayed are clinical characteristics, laboratory parameters, clinical scores, and for describing the severity of liver fibrosis in liver biopsy samples, a scoring system called the Ishak score was used, capturing features such as inflammation, fibrosis, and necrosis as predictor factors, of the entire patient cohort at the time of liver biopsy

*Abbreviations CI* Confidence interval, *HE* Hepatic encephalopathy, *HR* Hazard ratio, *HVPG* Hepatic venous pressure gradient, *ICU* Intensive care unit, *LDH* Lactate dehydrogenase, *MELD* Model for end stage liver disease, *RRT* Renal replacement therapy

### Impact of histopathological degree of intrahepatic necrosis on further secondary clinical endpoints

It was also investigated whether the histological extent of intrahepatic necrosis had a significant impact on further secondary clinical endpoints (Table 4). However, degree of intrahepatic necrosis was neither associated with later need for intubation and invasive ventilation, RRT or HU listing for LTX. Only consecutive ICU admission was more likely with higher extent of intrahepatic necrosis (Odds ratio (OR) 1.04 (95% Confidence Interval (CI) 1–1.08), p = 0.046). Another potential strength of transjugular liver biopsy (TJLB) may lie in its ability to exclude advanced fibrosis through histopathological analysis. This allows for definitive confirmation of acute liver failure (ALF) and exclusion of acute-on-chronic liver failure (ACLF).

**Table 4:** Impact of histopathological degree of intrahepatic necrosis on secondary clinical endpoints

	**OR**	**95% CI**	**p**
ICU admission	1.04	1 – 1.08	**0.046**
Intubation	1	0,99 – 1.06	0.2
RRT	1	1 – 1.05	0.6
HU listing for LTX	1.04	1 – 1.09	0.07

Displayed are secondary endpoints which are presented as univariate parameters such as

*Abbreviations*: *CI* Confidence interval, *HU* High-urgency, *ICU* Intensive care unit, *LTX* Liver transplantation, *OR* Odds ratio, *RRT* Renal replacement therapy

## Discussion

In this retrospective study, analysing TJLB in 43 patients with ALF over a period of ten years, TJLB was safely performed and specimens were representative for further analysis. However, the clinical value of the histopathological specimen obtained was limited in the majority of patients. In fact, the histopathological evaluation of TJLB confirmed the initially already clinically presumed aetiology of ALF in most of the patients without changing the therapeutic approach. Of note, a subset of patients with highly suspected aetiologies potentially responsive to immunosuppressive treatment (e.g. AIH, GvHD) represented an exception, as long-term immunosuppressive therapy could be commenced in these patients following definitive histopathological confirmation. Histopathological criteria obtained by TJLB, including the extent of intrahepatic inflammation or necrosis, did not add significant value in predicting later transplant-free survival nor further clinical endpoints such as the later need for intubation, vasopressors or renal replacement therapy when compared to clinical evaluation alone.

Several previous studies have investigated the value of TJLB in patients with ALF. How does this present study compare to these and what novel information does it add to the open question of the exact clinical significance of performing TJLB as part of the routine work up procedure of patients with acute liver failure?

First, major previous studies are partly dating back more than 30 years [10] therefore not taking into account the improved possibilities of serological diagnostics regarding clarification of the aetiology of acute liver failure (e.g. improved autoimmune hepatitis serology, viral serology and PCR testing) independent of liver biopsy.

Second, the majority of studies have not analyzed in what proportion the results of the biopsy in fact changed both the presumed aetiology and the subsequent therapeutic approach [10, 17–19]. Importantly, our study evaluated in each patient the distinct value of biopsy both in terms of change of presumed aetiology as well as subsequent therapeutic decision making when compared to presumed aetiology and therapeutic approach before performing the biopsy. The therapeutic approach was not influenced in the majority of patients in the present study, therefore questioning the distinct value of TJLB in the general workup of all patients with ALF. In a more recent Italian study of TJLB in ALF, diagnosis was changed in 17% of cases, but in all patients only in respect to previously unknown liver cirrhosis and not concerning ALF aetiology [18]. However, in a significant number of patients with a high suspicion for aetiologies potentially be treatable by medical immunosuppression (e.g. AIH and GvHD), biopsy confirmed definitive diagnosis of these aetiologies and therefore enabled commencement of longer-term immunosuppressive treatment, which is line with results reported previously in this specific patient sub-cohort of ALF patients [18, 20]. The significant impact of TJLB in this subset of patients appears to be particularly important as presence of autoimmune hepatitis (AIH) antibodies, although at general low titers, was common in about 60% of patients in this study, a well-known phenomenon observed in patients with ALF [21]. Thirty-five percent of patients in this study were considered for HU liver transplantation. Therefore, an additional potential advantage of TJLB may lie in the exclusion of higher-grade fibrosis by histopathological analysis then enabling definitive confirmation of ALF and exclusion of ACLF to path the way for high urgent (ALF) in contrast to a mere laboratory MELD driven (ACLF) listing for liver transplantation. It may be further worth mentioning that sickle cell fraction and not liver histology should be considered the major factor determining begin and continuation of exchange transfusions in patients with sickle cell disease.

Third, previous studies have suggested that degree of intrahepatic necrosis adds distinctive value in predicting inferior outcome, however regularly without including parameters of clinical reasoning alone [10, 18]. In the present analysis we therefore used multivariate Cox-proportional hazard regression modelling including clinical parameters such as the BilE Score. The histopathological findings in the present study showed a high prevalence of necrosis in the patients examined indicating the overall advanced stage of acute liver failure. Statistical analysis however showed neither significant correlation between extent of intrahepatic necrosis on transplant-free survival nor on further clinical endpoints such as need of intubation, vasopressors or RRT. This rather surprising finding underscores the potential of sampling errors due to small specimen size in TJLB. In contrast, a clinical score such as the BiLE score showed a significant influence on transplant-free survival in both uni- and multivariate analyses illustrating the high prognostic importance of clinical parameters such as lactate, bilirubin and aetiology in acute liver failure [15]. In line, a recent study found a close correlation of advanced mutlilobular necrosis on biopsy with higher INR, MELD and MELD-Na Scores [19]. Presence of multilobular necrosis was the sole independent predictor of increased mortality in this study, however without including lactate concentration or presumed ALF aetiology into the prediction model [19]. About 25% of patients in this present study received TPE as an adjunctive treatment measure, which has been demonstrated to improve LTX free survival in previous studies [22, 23], therefore potentially representing a confounding factor in respect to outcome prediction using histologic criteria that have been evaluated before implementation of TPE [10, 18].

Fourth, previous studies [10, 17–19] have almost exclusively analysed liver-transplant-free survival as a relevant endpoint that might be predicted by results of transjugular liver biopsy. In this manuscript, we therefore additionally analysed several further important clinical endpoints such as admission to the intensive care unit or the need for intubation, vasopressor support or renal replacement therapy in dependence of intrahepatic necrosis extent suggested by biopsy.

To our knowledge, there have been no fatal events reported following transjugular liver biopsy in more recent literature [19, 24, 25]. Although TJLB appeared to be safely performed in this and previous studies [19, 20] in ALF patients, it is still a resource intensive procedure as it cannot be executed in the ICU environment for lack of X-ray fluoroscopic capacity. The results of this study question the general need for TJLB to be performed routinely in all patients with ALF, as the therapeutic approach was regularly not changed in these patients and outcome prediction was not superior to clinical reasoning alone.

TJLB may provide a potential advantage in cases of AIH in relation to specific therapeutic options. However, all patients in this present study that were diagnosed with AIH either underwent LTX or died despite immunosuppressive therapy. Once patients with AIH present in ALF, aggressive immunosuppressive therapy may prove to be a double-edged sword. Firstly, once patients present with ALF, aggressive IS may not reverse the disease process any more [26], secondly patients may end up being at increased risk of sepsis which could jeopardize rescue therapies such as LTX.

This study has limitations, mainly its retrospective character and the single-center setting, limiting generalizability of the data. Due to the observative character of the study, only assumptions concerning potential impact of histopathological findings on clinical endpoints such as liver-transplant free survival can be drawn. Further, presumed diagnosis and therapeutic approaches before and after TJLB were analysed retrospectively from chart records and not prospectively, thus potentially suggesting a too high pre-test certainty of the presumed diagnoses. The question what histological findings suggest with reasonable certainty DILI is still under debate as one specific finding pointing towards DILI has not been identified yet due to the multitude of different injury agents and diverse histologic patterns associated with those [27, 28]. In this retrospective analysis, a predominant portal neutrophilic (and eosinophilic) infiltrate with at the same time absent portal and intra-acinar plasma cells, missing rosette formation and absent significant intrahepatic deposition of iron or copper were considered as compatible with potential DILI associated ALF. The correlation of degree of hepatic necrosis and laboratory indices of severity of liver failure is fraught with difficulty as there is a significant risk of sampling error considering selective biopsy of necrotic areas versus areas of regeneration.

In spite of this limitations, these systematic real-life data from a large liver transplant-center represent one of the largest analyses on use of TJLB in ALF. Further, the consideration of clinical and routine laboratory data together with histopathological findings concerning prognostication of multiple important clinical outcomes (not confined to liver-transplant free survival alone), enable a more realistic modelling than consideration of histopathological criteria alone. The comparison of both presumed aetiology and therapeutic approach before and after biopsy may allow for a more unbiased assessment of the clinical impact of this time and resource consuming procedure. Certainly, future prospective and optimal randomized studies would be desirable to further explore the value of routine TJLB in patients with ALF, desirable identifying certain subgroups that might benefit most from this procedure in terms of clinical decision making and outcome prediction.

## Conclusions

The present analysis demonstrated that the performance of TJLB in ALF was safe but led to a change in suspected diagnosis and to a significant change in therapeutic measures only in those patients with a presumed high risk for aetiologies potentially responsive to immunosuppressive therapy. Clinical assessment alone was accurate enough, with additional histopathological examination adding no significant value, to predict overall prognosis of patients with ALF. Overall, this study contributes to the discussion on the value of routinely performing TJLB in ALF patients. Future prospective studies need to clarify the role of TJLB in the diagnostic and therapeutic approach of ALF.

## Data Availability

The datasets used and analyzed are during the current study are available from the corresponding author on reasonable request.

## Abbreviations

ACLF: Acute on chronic liver failure
ALF: Acute liver failure
ANA: Antinuclear antibodies
BMI: Body mass index
CI: Confidence interval
Gy: Gray
HR: Hazard ratio
HVPG: Hepatic venous pressure gradient
HU: High urgency list
ICU: Intensive care unit
IQR: Interquartile range
LDH: Lactate dehydrogenase
LKM: Liver-kidney microsomal antibodies
LTX: Liver transplantation
MELD Score: Model of end stage liver disease Score
PDMS: Patient data monitoring system
RRT: Renal replacement therapy
SMA: Smooth-muscle antibodies
TJLB: Transjugular liver biopsy
TPE: Therapeutic Plasma Exchange
